# Comparable survival in rats with intracranial glioblastoma irradiated with single-fraction conventional radiotherapy or FLASH radiotherapy

**DOI:** 10.3389/fonc.2023.1309174

**Published:** 2024-01-16

**Authors:** Emma Liljedahl, Elise Konradsson, Karin Linderfalk, Emma Gustafsson, Kristoffer Petersson, Crister Ceberg, Henrietta Nittby Redebrandt

**Affiliations:** ^1^ The Rausing Laboratory, Division of Neurosurgery, Department of Clinical Sciences, Lund University, Lund, Sweden; ^2^ Medical Radiation Physics, Department of Clinical Sciences, Lund University, Lund, Sweden; ^3^ Department of Oncology, Medical Research Council Oxford Institute for Radiation Oncology, University of Oxford, Oxford, United Kingdom; ^4^ Radiation Physics, Department of Haematology, Oncology and Radiation Physics, Skåne University Hospital, Lund University, Lund, Sweden; ^5^ Department of Neurosurgery, Skåne University Hospital, Lund, Sweden

**Keywords:** glioblastoma, FLASH-RT, CONV-RT, tumor size, rat model

## Abstract

**Background:**

Radiotherapy increases survival in patients with glioblastoma. However, the prescribed dose is limited by unwanted side effects on normal tissue. Previous experimental studies have shown that FLASH radiotherapy (FLASH-RT) can reduce these side effects. Still, it is important to establish an equal anti-tumor efficacy comparing FLASH-RT to conventional radiotherapy (CONV-RT).

**Methods:**

Fully immunocompetent Fischer 344 rats with the GFP-positive NS1 intracranial glioblastoma model were irradiated with CONV-RT or FLASH-RT in one fraction of 20 Gy, 25 Gy or 30 Gy. Animals were monitored for survival and acute dermal side effects. The brains were harvested upon euthanasia and tumors were examined post mortem.

**Results:**

Survival was significantly increased in animals irradiated with CONV-RT and FLASH-RT at 20 Gy and 25 Gy compared to control animals. The longest survival was reached in animals irradiated with FLASH-RT and CONV-RT at 25 Gy. Irradiation at 30 Gy did not lead to increased survival, despite smaller tumors. Tumor size correlated inversely with irradiation dose, both in animals treated with CONV-RT and FLASH-RT. Acute dermal side effects were mild, but only a small proportion of the animals were alive for evaluation of those side effects.

**Conclusion:**

The dose response was similar for CONV-RT and FLASH-RT in the present model. Tumor size upon the time of euthanasia correlated inversely with the irradiation dose.

## Introduction

Glioblastoma is extremely difficult to treat (1). Radiotherapy (RT) is one of the few treatment options that has led to increased survival, with a doubled survival time in irradiated patients compared to those who had surgery as stand-alone therapy (1). However, the dose of irradiation is limited by unwanted side effects on normal tissue. In the brain normal tissue damage is especially problematic due to cognitive side effects (2, 3). In order to reduce side effects, RT is usually delivered fractionated over a period of several weeks. The primary treatment option typically involves irradiation at a total dose of 60 Gy divided into 30 fractions concomitant with Temozolamide, followed by Temozolamide as adjuvant treatment after finished irradiation (4, 5). Still, survival is very short in patients with glioblastoma, with a median survival of just 12 months (6).

It would be beneficial to optimize radiotherapy against glioblastoma. Firstly, tumor control could potentially be increased if the total dose could be increased without severe side effects (7). Secondly, it seems like radiotherapy can elicit an immunologically mediated anti-tumor response (5, 8, 9), but the dose per fraction needs to be altered in order to elicit a more effective immunological response (10).

In order to try to reduce unintended side effects in the RT setting, FLASH-RT has been investigated. For example, the effects of FLASH-RT have been examined in different pre-clinical brain models and there is evidence to suggest that it may be possible to achieve a sparing effect on normal tissue (11–13). In juvenile mice, FLASH-RT irradiation at 8 Gy x 1 resulted in a better long-term cognitive function compared to CONV-RT at 8 Gy x 1 (14). Still questions remain, including:

- whether there is an equal anti-tumor efficacy of FLASH-RT compared to CONV-RT in fully immunocompetent animals with glioblastoma.
- if the same effects of FLASH-RT can be demonstrated in different models of glioblastoma.

In our previous work, we have demonstrated that FLASH-RT has equal anti-tumor efficacy as CONV-RT against glioblastoma cells implanted subcutaneously in fully immunocompetent animals (13). We have also demonstrated that both FLASH-RT and CONV-RT can mediate long-term immunity in fully immunocompetent rats (9).

In the present study, we wanted to evaluate if we could establish an effective irradiation dose in animals with intracranial glioblastoma irradiated with CONV-RT versus FLASH-RT in one fraction. In previous studies, we prolonged survival but did not achieve long-term cure after irradiation at the dose 12.5 Gy x 2 in animals with intracranial glioblastoma (9). In the present study, irradiation was delivered at single doses of 20 Gy, 25 Gy and 30 Gy. Optimal fractionation has not yet been extensively studied when it comes to FLASH-RT irradiation. Hence, we decided to first establish an optimal dose with one fraction. We chose our NS1 glioblastoma model, as it generates stable tumors in all control animals, and has an infiltrative growth pattern with perivascular spread, which renders it a good model of glioblastoma (13, 15, 16). Furthermore, the NS1 model is positive for green fluorescent protein, GFP, which means that it is easy to track even small tumor satellites with anti-GFP staining (15). Finally, since we have used the same model in subcutaneous trials, we wanted to develop the protocol further in the intracranial setting.

In the present study, the aim was to explore the following:

- Does single-fraction irradiation with FLASH-RT and CONV-RT have equal anti-tumor efficacy against intracranial glioblastoma (primary aim)?
- Does tumor size differ between animals depending on irradiation dose (secondary aim)?
- Are there any differences between FLASH-RT and CONV-RT when it comes to dermal side effects (tertiary aim)?

## Materials and methods

### Ethical considerations

The study was approved by the Regional Ethics Board in Lund-Malmö, Sweden (ethical permission number 1469-2022).

### Animals

Fully immunocompetent Fischer 344 rats were used (Fischer Scientific, Germany) as previously described by us (9, 17). The rats were housed in pairs in rat cages with water and rat chow *ad libitum*. The animals were monitored daily, and those displaying signs of paresis, epilepsy, or declined general condition, were euthanized with CO2 inhalation according to the ethical permission. All efforts were made to minimize animal suffering. Inoculations were performed under general anesthesia with isoflurane inhalation. All animals were randomized to their respective treatment groups at the initiation of the study.

### Tumor cell line

The rat GFP-positive glioblastoma tumor cell line NS1 was used to generate intracranial tumors. The tumors are IDH-wild type (IDH-wt) and exhibit necrosis and vascular proliferation. The NS1 cell line has been developed as previously described by us and it has been demonstrated that the tumors grow infiltratively, with perivascular dissemination (15).

### Tumor cell inoculations

NS1 cells were prepared for inoculation as previously described by us (9). In brief, cells were seeded in IMDM culture media with 10% fetal bovine serum, 1% sodium-pyruvate (100mM), 1% Glutamax (100X) and 0.05 mg/ml gentamycin (all from Gibco), and cultured in 37°C and 5% CO_2_ in a humidified incubator. Trypsin (Invitrogen) was added, and cells were incubated to detach the adherent cells. Cells were centrifuged at 1200 rpm for 5 minutes at 4°C, and then the supernatant was removed. Afterwards the cell pellet was re-suspended in serum-free medium.

In order to establish intracranial tumors, each rat received 5000 NS1 cells, suspended in 5 µl of PBS. Intracerebral tumor cell inoculation was done under isoflurane inhalation anaesthesia at 3-5% concentration of the gas mixed with air, using a stereotactic frame and a 10 µl Hamilton syringe. The cells were injected on the right side of the cranium at a depth of 5 mm, 2 mm laterally from the sagittal suture, and 1 mm anterior to the coronal suture. The cranial burr hole was sealed with bone wax, and the incision was closed with absorbable suture. Tumor cell inoculations were performed on day 0 in this experimental set-up.

### Radiotherapy

The animals were irradiated using a 10 MeV electron beam of a clinical linear accelerator (Elekta Precise, Stockholm, Sweden) operated with a pulse width of 3.5 µs and a pulse repetition frequency of 200 Hz. Before irradiation, animals were anaesthetized with intraperitoneal injection of Ketalar/Rompun and fixed in PMMA boxes. The boxes were positioned in a 1x1 cm^2^ irradiation field and the tumors were targeted using the crosshair of the linear accelerators light field with the cell inoculation site as a reference point.

Radiotherapy was administered at day 7 as a single fraction of either 20, 25, or 30 Gy, using CONV-RT or FLASH-RT. The average dose rate of the CONV-RT was 8 Gy/min. For FLASH-RT, the linear accelerator was temporarily modified to enable ultra-high dose rate electron delivery.

The absorbed dose was prescribed at 5 mm depth, i.e., at the same depth as the tumor cells were injected. The dose-per-pulse (for FLASH-RT) and dose-per-monitor unit (for CONV-RT) in this position was determined using radiochromic film (GafChromic EBT-XD, Ashland Advances Materials, Bridgewater NJ) at 5 mm depth in a polysterene phantom placed in one of the boxes. Film measurements were repeated prior to each treatment session to verify the delivered dose. During administration of FLASH-RT, a Farmer-type ionization chamber was used for relative output measurements to ensure output stability in FLASH-RT mode.

### Survival

**Figure 1:**
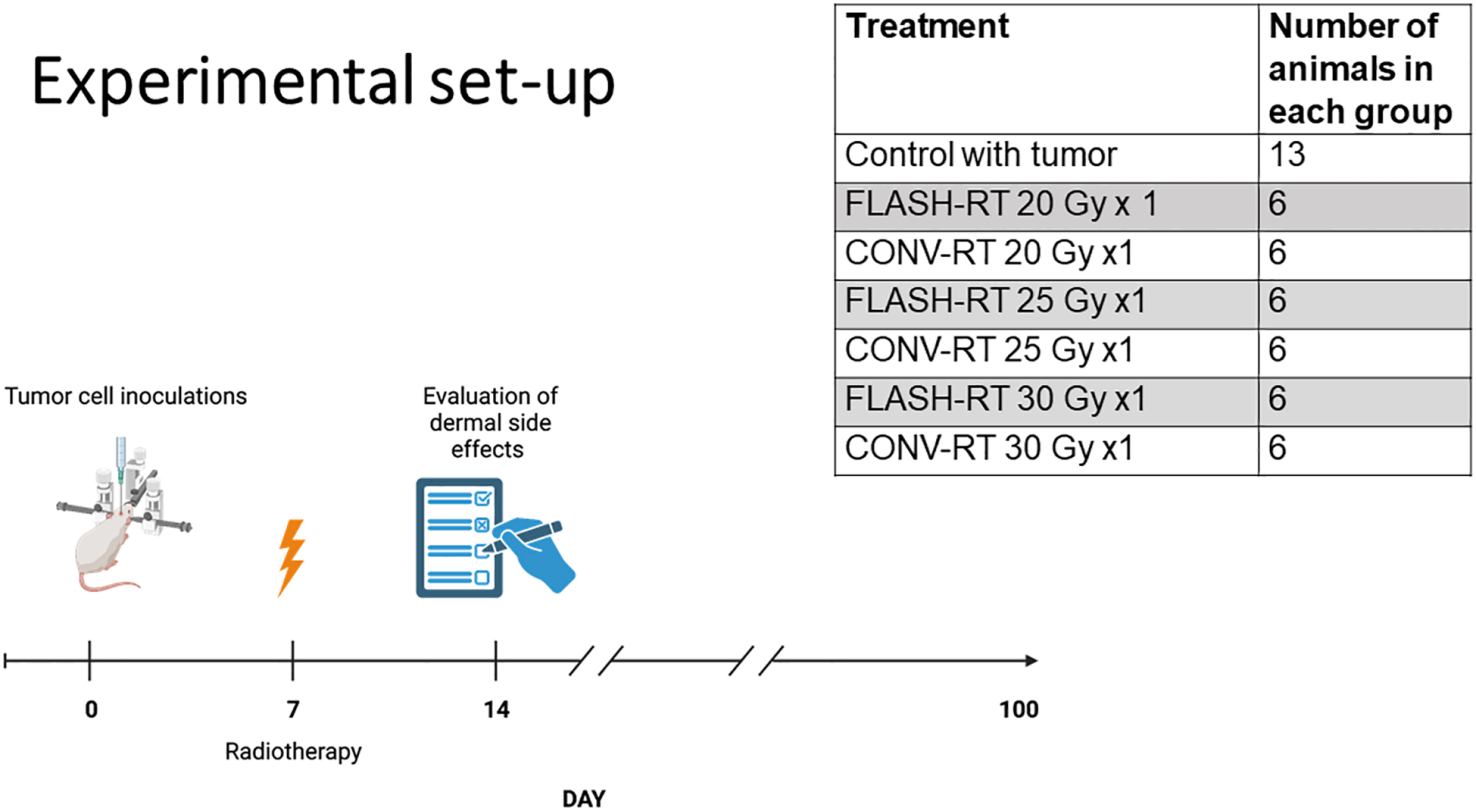
Experimental setup. Image created with Biorender.

Animals were monitored daily, and overall survival was determined as the number of days from inoculation (day 0) until the criteria for euthanasia were reached. Forty-nine animals were included in the survival study (**Figure 1**). The study endpoint was reached at day 100 after tumor inoculation.

### Dermal side effects

Animals were evaluated for acute radiation-induced skin reactions at two weeks after irradiation according to a phenotypic grading scale 1-6 (1: normal, 2: hair loss, 3: erythema, 4: dry desquamation, 5: <30% moist desquamation, and 6: >30% moist desquamation) established by de Andrade and colleagues (18) and previously used by us (13). Additionally, dermal side effects were evaluated at day 100 after irradiation.

### Histopathology

After euthanasia, brains were removed and fixed in 4% formaldehyde (HistoLab) for a minimum of 24 hours, followed by paraffin embedding and microtome sectioning of the samples to 7µm thickness. Brain samples were stained using a primary biotinylated goat anti-GFP antibody (ABIN1000087, antibodies-online), a VECTASTAIN® ABC-HRP goat IgG kit (PK-4005, Vector Laboratories), and a DAB detection kit (Agilent Dako). Mayers HTX (HistoLab) was used as a counterstain. Samples were mounted on microscope slides using Pertex® mounting media (HistoLab). Tumor size was analyzed with light microscopy, blinded to the treatment situation. A semi-quantitative score was used, comparing tumor size as demonstrated with anti-GFP staining to the whole brain cross-sectional area. The tumor size was assigned with 0=no visible tumor; 1=small tumor satellites; 2=intermediate size of a tumor that occupies less than half of the hemisphere in maximal size; 3=large size of a tumor that occupies more than half of the hemisphere in maximal size. The brains were investigated both frontally, at the site of the coronal suture, and posteriorly.

### Statistics

SPSS® was used for statistical calculations. Survival was tested with Log-Rank Mantel-Cox, with p < 0.05 for statistical significance. Fisher’s exact two-sided test was used for comparison between the groups regarding tumor size. Spearman’s two-sided test was used for evaluation of correlation.

## Results

### Dose delivery

Based on film measurements and the ion chamber signal, the agreement between the prescribed dose and the estimated delivered dose was within 4% for FLASH-RT and 1% for CONV-RT irradiations. FLASH-RT was delivered in 10, 12, or 15 pulses with mean dose-per-pulse values of 2.0-2.1 Gy, average dose rates ≥429 Gy/s, pulse dose rates ≥0.57x10^6 Gy/s, and total treatment times ≤70 ms (**Table 1**).

**Table 1:** The number of delivered pulses, dose-per-pulse, treatment times, and dose rates for FLASH-RT delivery of 20, 25, and 30 Gy, respectively.

Dose (Gy)	# pulses	Dose-per-pulse (Gy)	Treatment time (ms)	Average dose rate (Gy/s)	Pulse dose rate (Gy/s)
**20**	10	2.0	45	444	0.57 x 10^6^
**25**	12	2.1	55	455	0.60 x 10^6^
**30**	15	2.0	70	429	0.57 x 10^6^

### Survival

**Figure 2:**
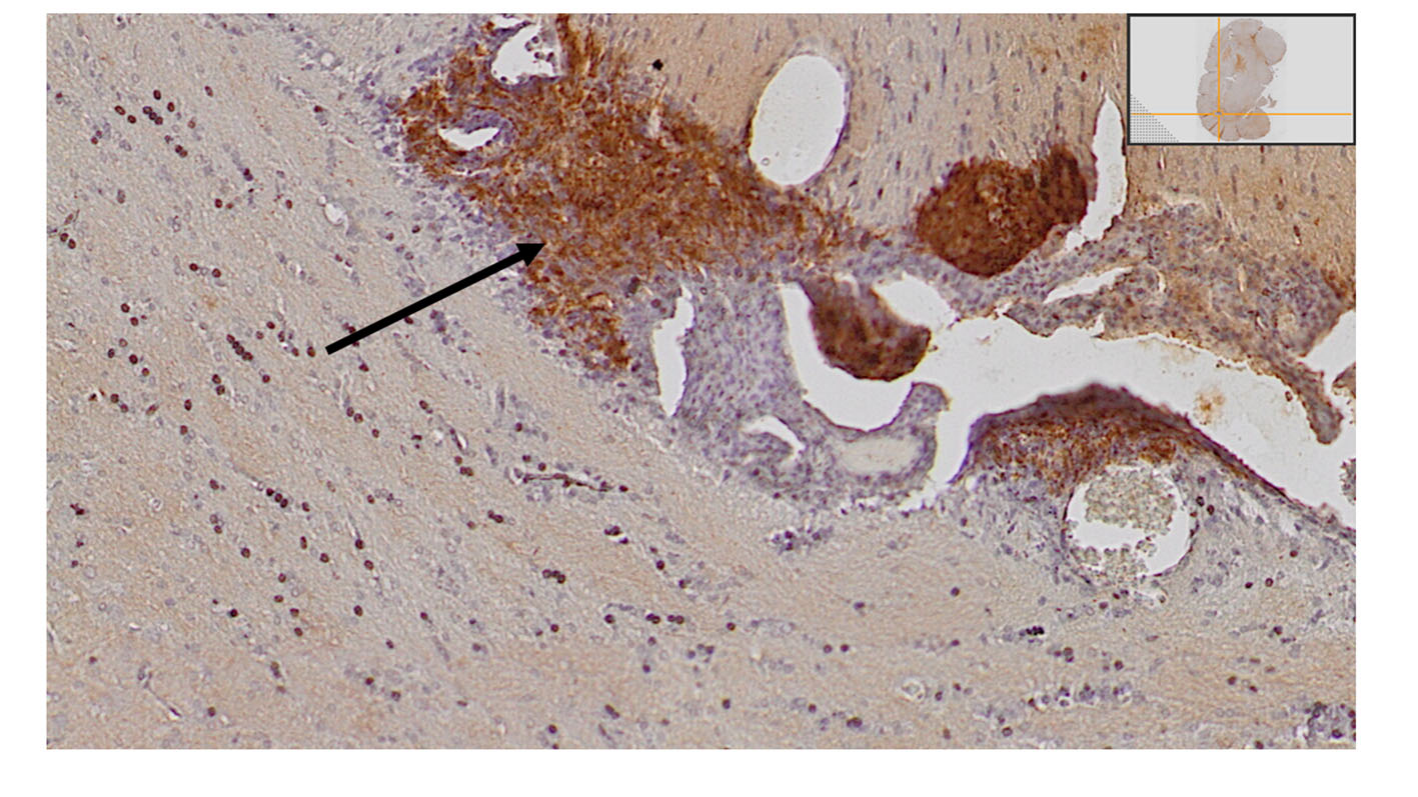
Small tumor satellites were detected in all animals that were euthanized on day seven after inoculations, as demonstrated with htx-staining and anti-GFP antibodies.

We delivered irradiation seven days after tumor cell inoculations. Apart from the forty-nine animals included in the survival study, we euthanized four additional animals on day 7 after tumor cell inoculations, but without any further treatment, and we evaluated the brains for tumor size. They all had detectable small tumors (**Figure 2**).

**Figure 3:**
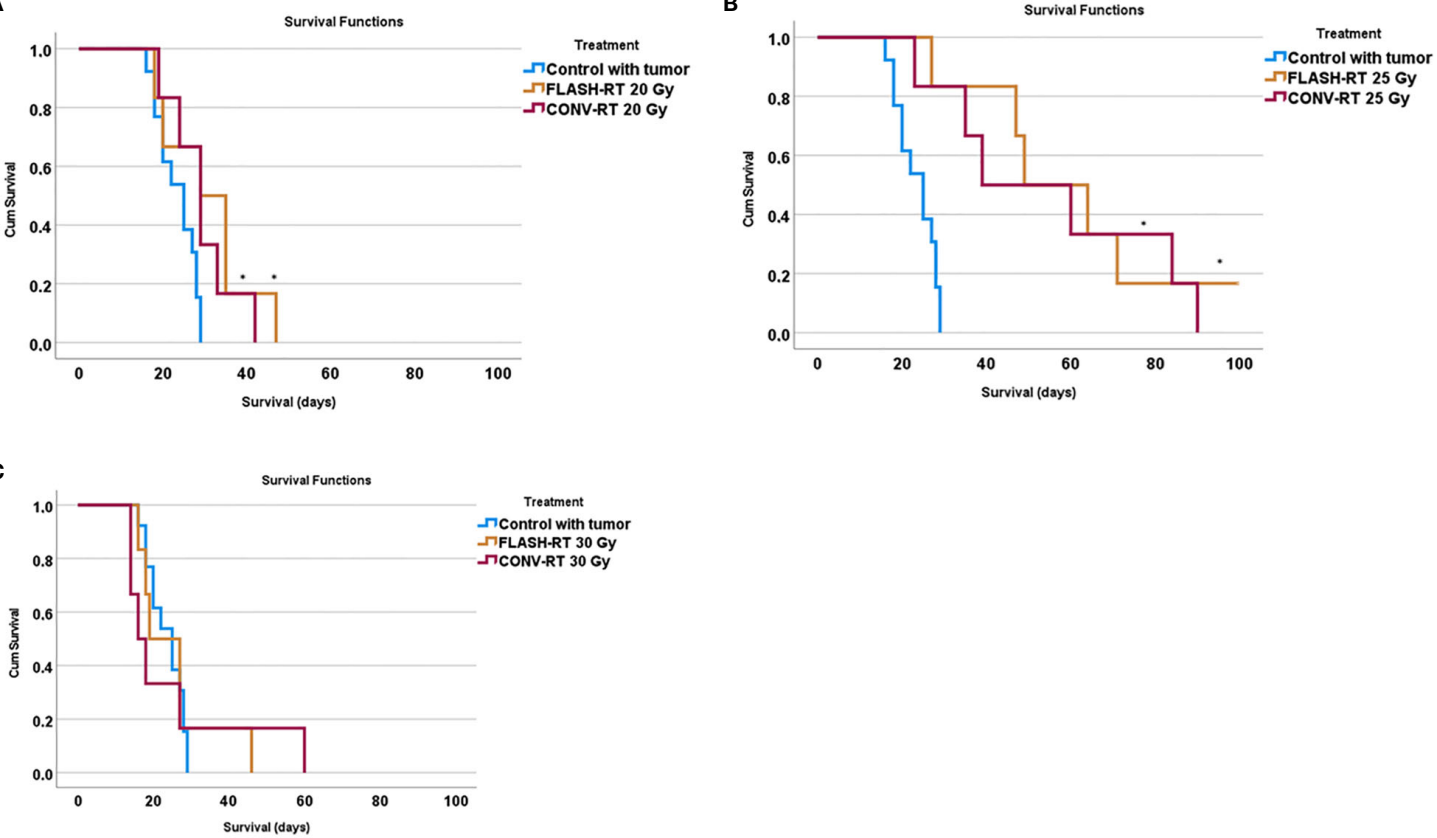
Survival curves in animals irradiated with FLASH-RT or CONV-RT at **(A)** 20 Gy, **(B)** 25 Gy and **(C)** 30 Gy compared to control animals. * indicates a significantly increased survival compared to control animals with p < 0.05.

We observed increased survival in animals irradiated with FLASH-RT 20 Gy as well as CONV-RT 20 Gy compared to control animals (FLASH-RT 20 Gy versus control p = 0.030; CONV-RT 20 Gy versus control p = 0.041, Log-Rank Mantel-Cox) (**Figure 3A**). We also saw a significantly increased survival in animals irradiated with FLASH-RT 25 Gy as well as CONV-RT 25 Gy compared to control animals (FLASH-RT 25 Gy versus control p = 0.001; CONV-RT 25 Gy versus control p= 0.002, Log-Rank Mantel-Cox) (**Figure 3B**). However, we did not see an increased survival in animals irradiated with FLASH-RT 30 Gy or CONV-RT 30 Gy compared to control animals (FLASH-RT 30 Gy versus control p > 0.05; CONV-RT 30 Gy versus control p > 0.05, Log-Rank Mantel-Cox) (**Figure 3C**, **Table 2**).

**Table 2:** Mean overall survival across the groups.

Treatment group	Mean ± SD (days)
Control	23 ± 5
FLASH-RT 20 Gy	31 ± 11
FLASH-RT 25 Gy	60 ± 25
FLASH-RT 30 Gy	26 ± 11
CONV-RT 20 Gy	29 ± 8
CONV-RT 25 Gy	55 ± 50
CONV-RT 30 Gy	25 ± 18

Our longest observed overall survival was reached in the animals irradiated with FLASH-RT at 25 Gy, with a mean overall survival of 60 days, followed by those irradiated with CONV-RT 25 Gy with a mean overall survival of 55 days (**Table 2**).

The power to detect a significant survival at the 0.05 significance level was 0.939.

### Tumor size

**Figure 4:**
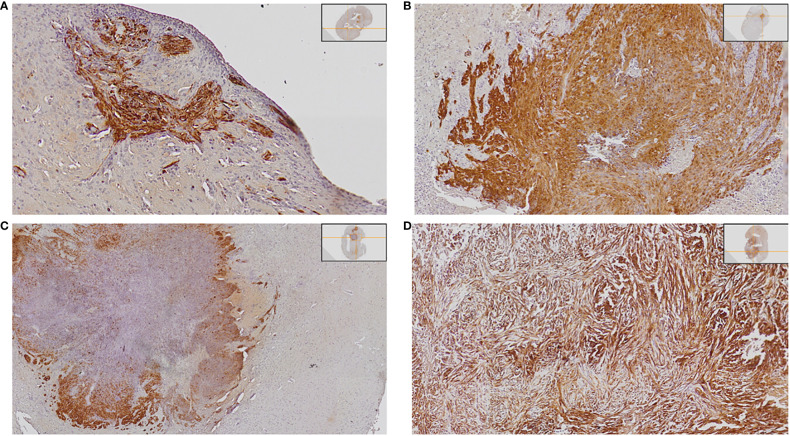
Demonstration of different tumor sizes. The tumor is detected with anti-GFP staining visualized with DAB, and nuclei are stained with hematoxylin. Examples are presented below: **(A)** Detection of small tumor satellites (semi-quantitative score 1). **(B)** Intermediate size of tumor occupying < half of one hemisphere (semi-quantitative score 2). **(C)** Intermediate size of tumor occupying < half of one hemisphere (semi-quantitative score 2). **(D)** Large tumor with a mass effect on surrounding brain tissue (semi-quantitative score 3).

We harvested and examined the brains in relation to tumor size upon euthanasia. Tumor size was evaluated with histopathological examinations using the semi-quantitative score of anti-GFP staining in relation to the whole-brain cross-sectional area. Representative images are presented in **Figure 4**. We could not analyze three brains (two irradiated with CONV-RT and one control) for technical reasons.

**Figure 5:**
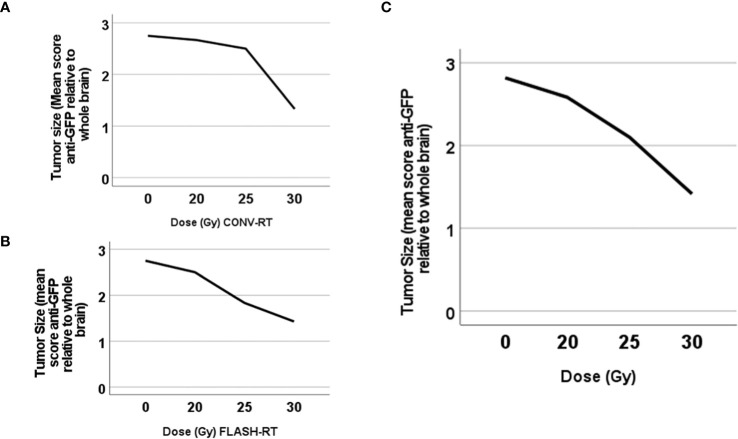
Tumor size differed significantly between animals irradiated at different dose levels. Plot of the tumor size (mean size in each group) according to the semi-quantitative score in relation to irradiation dose in animals irradiated with **(A)** CONV-RT or **(B)** FLASH-RT or **(C)** either CONV-RT or FLASH-RT.

Control animals with tumors but no further treatment all had tumors, of intermediate size or large size (**Figures 5A–C**, **Table 3**). Tumor size differed significantly between the groups upon euthanasia (Fisher’s exact 2-sided test p = 0.001). Since the tumors were positive for GFP, even small satellites were easily detected with anti-GFP antibody staining, as demonstrated in **Figure 4A**. No animal treated with CONV-RT 30 Gy or FLASH-RT 30 Gy presented with large tumors occupying more than half of the hemisphere upon euthanasia. In one animal irradiated with FLASH-RT at 25 Gy, that survived 100 days, we could not detect any tumor at the end of the study. Another animal that had been irradiated with FLASH-RT at 25 Gy died on day 49, with no visible signs of any large tumor. The other animals irradiated with FLASH-RT at 25 Gy or CONV-RT at 25 Gy had intermediate or large tumors (**Table 3**).

**Table 3:** Tumor size upon euthanasia. Mean tumor size is reported according to the semi-quantitative score.

Treatment	No visible tumor (score 0)	Detection of satellites, no tumor bulk (score 1)	Intermediate tumor < half hemi-sphere (score 2)	Large tumor > half hemi-sphere (score 3)	Mean tumor size
Control with tumor	0	0	3	9	2.75
FLASH-RT 20 Gy	0	1	1	4	2.50
FLASH-RT 25 Gy	2	0	1	3	1.83
FLASH-RT 30 Gy	0	3	3	0	1.50
CONV-RT 20 Gy	0	0	2	4	2.67
CONV-RT 25 Gy	0	0	2	2	2.50
CONV-RT 30 Gy	0	4	2	0	1.33

Next, we tested tumor size for correlation with delivered irradiation dose, with a significant negative correlation between tumor size and irradiation dose when all animals were included (Spearman’s coefficient -0.61, p=0.000).

### Dermal side effects

**Figure 6:**
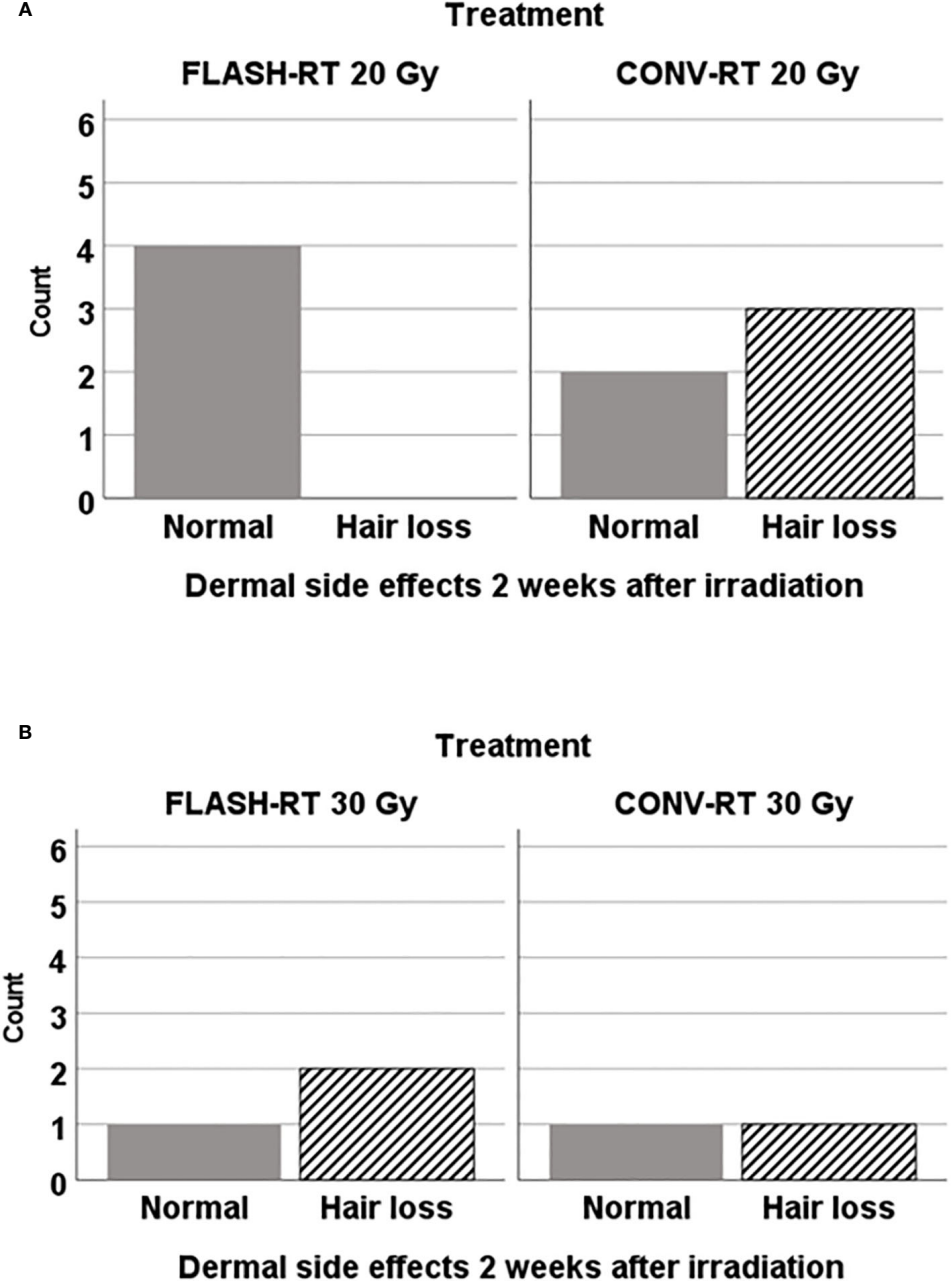
Dermal side effects were evaluated two weeks after irradiation in animals irradiatied with FLASH-RT or CONV-RT at **(A)** 20 Gy x 1 (n=9 animals alive) and **(B)** at 30 Gy x 1 (n=5 animals alive). Only those animals irradiated with FLASH-RT 20 Gy x1 were spared from any sign of dermal side effects at that time point.

We evaluated dermal side effects two weeks after irradiation in animals irradiated with 30 Gy or 20 Gy. Dermal side effects were mild, and only consisted of hair loss in some of the animals. We observed that FLASH-RT irradiation at 20 Gy led to no dermal side effects, in contrast to CONV-RT at the same irradiation dose we observed hair loss. However, as demonstrated in **Figure 6**, only a minor proportion of all the animals were alive at that time point, meaning that the results should be interpreted with care. Unfortunately, due to the small remaining sample size in each group, we could not perform an evaluation at day 100 after tumor inoculations.

## Discussion

In the present study, we demonstrated that a single fraction of CONV-RT versus FLASH-RT resulted in comparable survival in fully immunocompetent animals with intracranial glioblastoma. At 20 Gy x 1 and 25 Gy x 1, CONV-RT and FLASH-RT resulted in significantly increased survival compared to unirradiated control animals. Increasing the dose further to 30 Gy x 1, however, did not increase survival significantly compared to control animals even though none of the animals irradiated with 30 Gy x 1 died with a massive tumor burden, suggesting that the irradiation dose had been too high in relation to the acute radiotoxic effects that the animals could tolerate. Overall mean survival was longest in animals irradiated with FLASH-RT at 25 Gy x 1, followed by those irradiated with CONV-RT at 25 Gy x 1.

Our glioblastoma model is based on fully immunocompetent animals, with an aggressive intracranial tumor. All control animals that received tumor cell inoculations but no further treatment, died due to tumor growth. In this study, we analyzed the brains of the animals with immunohistochemistry in order to establish if the animals died *due to* large intracranial tumor burden, or if the animals died *with* just a small tumor. Since the tumors in the present study express GFP, we could easily track tumor growth with anti-GFP immunostaining, even when the tumors are small satellites. Animals that had been irradiated with 30 Gy with FLASH-RT or CONV-RT, did not die with large tumors, but indeed the majority only had small tumor satellites. This differed from those irradiated with 20 Gy, where the majority of the animals died with massive tumors, which indicates that the dose of 20 Gy x 1 was too low to achieve sufficient tumor control. Indeed, we demonstrated a negative correlation between tumor size upon euthanasia and irradiation dose.

There are several hypotheses covering the mechanisms behind radiation-induced damage in the brain (7, 19). Neuro-inflammation takes place following irradiation, and depending on the dosage and fractionation, this may lead to chronically elevated oxidative stress (7). Mature neurons might survive irradiation, but still undergo a change in their transmitting abilities. In the hippocampus, irradiation can lead to long-term loss of dendritic spines (7). Furthermore, irradiation induced damage to the blood-brain barrier, due to alterations of the microvasculature, can lead to ischemia and neuro-toxicity (7). It has been demonstrated that irradiation with FLASH-RT led to reduced reactive gliosis in the brain of mice compared to CONV-RT (20). In the present study we did not explore the mechanisms behind the radiation-induced effects and side effects. This is an important focus for further study - exploring immunohistochemical alterations and gene expression changes at set doses and time points.

Dermal side effects are important in relation to wound healing complications due to radiotherapy since acute reactions might lead to haltering of the irradiation in the clinical setting (21). Also, radiation ulcers and infections can lead to long-term problems (21). In glioblastoma patients, it has been suggested that early initiation of radiotherapy might increase the risk for surgical site infections due to wound healing problems (22). With the early evaluation of dermal side effects already two weeks after irradiation, acute dermal side effects were covered. It turned out that even in the case of irradiation at the area of the skin incision only seven days after surgery, no severe dermal side effects were detected in our present study, regardless of the modality used for radiotherapy. It would have been interesting to evaluate dermal side effects in a longer time perspective if a large proportion of the animals had been alive at the end of the experiment. According to our previous research on dermal side effects in relation to irradiation with FLASH-RT or CONV-RT, no difference could be seen in the subcutaneous tumor setting one to four weeks after irradiation with 15 Gy x 3, as well as in the long-term perspective of three months post irradiation (13).

Other studies have shown a cognitive sparing effect of FLASH-RT in relation to CONV-RT (23–25). In future studies, it would be interesting also to explore the effects of fractionation and dose rate in relation to dose-response and cognitive side effects. Others have demonstrated that sparing effects on long-term potentiation is achieved by fractionation of FLASH-RT in doses of 3 Gy x 10, as compared to CONV-RT at the same dosage (26). We would like to explore cognitive and dermal side effects, comparing FLASH-RT and CONV-RT, but in order to get a better statistical power in long-term survivors, an irradiation dose resulting in an even higher degree of survival would be needed.

## Conclusions

We demonstrated that single-fraction CONV-RT and FLASH-RT are equally effective against intracranial glioblastoma in the fully immunocompetent rat model of the present study. Animals irradiated at the highest dose level of 30 Gy x 1, did not die due to massive intracranial tumor burden. Those irradiated at the lower dose level of 20 Gy x 1 or control animals on the other hand, had a larger tumor burden upon euthanasia. With the model used in this study, we demonstrated that tumor size correlated inversely with irradiation dose.

## Data availability statement

The raw data supporting the conclusions of this article will be made available by the authors, without undue reservation.

## Ethics statement

The animal study was approved by Regional Ethics Board in Lund-Malmö, Sweden. The study was conducted in accordance with the local legislation and institutional requirements.

## Author contributions

EL: Conceptualization, Data curation, Formal analysis, Investigation, Methodology, Visualization, Writing – original draft. EK: Conceptualization, Data curation, Formal analysis, Investigation, Methodology, Writing – review & editing. KL: Conceptualization, Data curation, Formal analysis, Investigation, Methodology, Project administration, Visualization, Writing – original draft. EG: Data curation, Investigation, Project administration, Writing – original draft. KP: Conceptualization, Supervision, Writing – original draft. CC: Conceptualization, Data curation, Formal analysis, Funding acquisition, Investigation, Methodology, Resources, Software, Supervision, Validation, Writing – original draft. HN: Conceptualization, Data curation, Formal analysis, Funding acquisition, Investigation, Methodology, Project administration, Resources, Supervision, Validation, Visualization, Writing – original draft.
